# Spray drift evaluation with point clouds data of 3D LiDAR as a potential alternative to the sampling method

**DOI:** 10.3389/fpls.2022.939733

**Published:** 2022-07-18

**Authors:** Longlong Li, Ruirui Zhang, Liping Chen, Boqin Liu, Linhuan Zhang, Qing Tang, Chenchen Ding, Zhen Zhang, Andrew J. Hewitt

**Affiliations:** ^1^Research Center of Intelligent Equipment, Beijing Academy of Agricultural and Forestry Sciences, Beijing, China; ^2^National Research Center of Intelligent Equipment for Agriculture, Beijing, China; ^3^National Center for International Research on Agricultural Aerial Application Technology, Beijing, China; ^4^College of Mechanical and Electronic Engineering, Northwest A & F University, Yangling, China; ^5^College of Information Science and Engineering, Shandong Agricultural University, Taian, China; ^6^Centre for Pesticide Application and Safety, The University of Queensland, Brisbane, QLD, Australia

**Keywords:** plant protection, droplet, spray drift, point clouds, 3D LiDAR, remote sensing

## Abstract

Spray drift is an inescapable consequence of agricultural plant protection operation, which has always been one of the major concerns in the spray application industry. Spray drift evaluation is essential to provide a basis for the rational selection of spray technique and working surroundings. Nowadays, conventional sampling methods with passive collectors used in drift evaluation are complex, time-consuming, and labor-intensive. The aim of this paper is to present a method to evaluate spray drift based on 3D LiDAR sensor and to test the feasibility of alternatives to passive collectors. Firstly, a drift measurement algorithm was established based on point clouds data of 3D LiDAR. Wind tunnel tests included three types of agricultural nozzles, three pressure settings, and five wind speed settings were conducted. LiDAR sensor and passive collectors (polyethylene lines) were placed downwind from the nozzle to measure drift droplets in a vertical plane. Drift deposition volume on each line and the number of LiDAR droplet points in the corresponding height of the collecting line were calculated, and the influencing factors of this new method were analyzed. The results show that 3D LiDAR measurements provide a rich spatial information, such as the height and width of the drift droplet distribution, etc. High coefficients of determination (*R*^2^ > 0.75) were observed for drift points measured by 3D LiDAR compared to the deposition volume captured by passive collectors, and the anti-drift IDK12002 nozzle at 0.2 MPa spray pressure has the largest *R*^2^ value, which is 0.9583. Drift assessment with 3D LiDAR is sensitive to droplet density or drift mass in space and nozzle initial droplet spectrum; in general, larger droplet density or drift mass and smaller droplet size are not conducive to LiDAR detection, while the appropriate threshold range still needs further study. This study demonstrates that 3D LiDAR has the potential to be used as an alternative tool for rapid assessment of spray drift.

## Introduction

Pesticides have significantly contributed to global agricultural development and food supplies (Oerke, 2006). Pesticide application is affected by complex environmental factors (e.g., temperature, humidity, and wind speed) and application techniques (Hilz and Vermeer, 2013). Consequently, it is estimated that 30% to 50% of the applied product drifts into non-target areas (Berg et al., 1999). Spray drift is defined by the US Environmental Protection Agency (EPA) as “the physical movement of a pesticide through the air at the time of application or soon thereafter, to any site other than the one intended for application” (EPA-United States Environmental Protection Agency, 2018). Spray drift is one of the largest sources of pollution caused by pesticides and poses significant risks to human health and the environment (Zhang et al., 2018). Many studies have investigated the effects of spray drift on ecosystems (Jong et al., 2008), water (Zhang et al., 2017), agriculture workers (Schampheleire et al., 2007), and exposure to bystanders and residents (Tsakirakis et al., 2018). Pesticides travel thousands of kilometers through air currents, eventually reaching remote areas (Stoughton et al., 1997). With the increasing awareness of the need for environmental protection, spray drift during pesticide application has attracted significant research attention globally.

Several factors such as meteorological conditions, application techniques, spray characteristics, spray equipment, target crops, and operator skills affect the degree of spray drift (Gil and Sinfort, 2005; Heidary et al., 2014). Regarding the application technology, the droplet size is widely recognized as the main factor affecting spray drift (Elliott and Wilson, 1983), and the effects of nozzle type, nozzle size, spray pressure, and additives on droplet size characteristics have been explored (Taylor et al., 2004; Nuyttens et al., 2007a). In addition, to reduce spray drift, components such as air-inclusion nozzles and low-drift nozzles with preset orifice settings have been designed to increase the droplet size (Butler Ellis et al., 2002). These specially designed nozzles can be used in harsh environments with a higher wind speed and dry conditions.

Before pesticide spraying, it is necessary to understand the anti-drift performance of the nozzle to facilitate the selection of the most appropriate nozzle (Ru et al., 2014). Based on ASAE Standard 572.1: 2009, the droplet size is divided into six classes—very fine, fine, medium, coarse, very coarse, and extra coarse (ASAE, 2009). The nozzle spray drift is commonly tested either in the field or in a wind tunnel. Field tests are complex, cumbersome, and costly, with specific requirements for the testing site and environmental stability. ISO 22866:2005 (ISO, 2005) specifies the procedures for conducting field tests, but this requires several people to work collaboratively. A series of experiments can take several hours to complete, with extremely high environmental crosswind requirements. If the wind direction changes more than 30° during the test, the measurement line must be reset (Arvidsson et al., 2011a). Wind tunnel tests were introduced to evaluate the spray drift characteristics (Southcombe et al., 1997; Herbst, 2001) by artificially controlling the temperature, humidity, wind speed, and wind direction to understand the influence of a single factor (Nuyttens et al., 2009; Zhang et al., 2015; Ferguson et al., 2016). ISO 22856:2008 standardizes the procedure for wind tunnel drift measurement (ISO, 2008).

For field and wind tunnel tests, sampling methods are mostly adopted to measure spray drift. Passive collectors, such as filter paper (Nuyttens et al., 2007b), plastic card (Carlsen et al., 2006), Petri dishes (Caldwell and Wolf, 2006), polyethylene line (Bai et al., 2013), nylon rope (Bui et al., 1998), dynamic rotating sampler (Bonds et al., 2009), and isokinetic sampler (Arvidsson et al., 2011b), were used for receiving the drift droplets, and the amount of spray deposition is quantified by discrete sampling. Each test cycle takes a long time to complete, as this method involves multiple processes, such as sample arrangement, collection, elution, and instrumental analysis. Furthermore, it is difficult to determine the spatial dispersion and evolution of spray drift clouds by point measurements. Therefore, new spray drift detection techniques or devices have been proposed and tested to develop easy and efficient alternative methods. Simulations of the transport process of spray droplets have been conducted, forming drift prediction models, such as AGDISP (Forster et al., 2012), AgDRIFT (Teske et al., 2000), RTDrift (Lebeau et al., 2011), and VALDRIFT (Allwine et al., 2010). Other studies have developed regression equations considering meteorological conditions and the drift distance to provide a reference point for the selection of nozzles and additives (Zhang et al., 2015). In addition, a mass balance system (Balsari et al., 2005) and test bench (Balsari et al., 2007) for drift measurement in an orchard and boom spraying have been developed, and were applied to measure spray drift of different types of nozzles (Gil et al., 2014; Grella et al., 2019).

With recent developments in sensor technology, the use of non-contact sensors for evaluating spray drift has become a trend. Many studies have been conducted using laser imaging (Wang et al., 2019), infrared thermal imaging (Jiao et al., 2016), and OP-FTIR (Kira et al., 2018) to assess spray drift. Compared with direct sampling, sensor detection reduces time and labor cost, providing information on the spatial variation of spray drift. Light detection and ranging (LiDAR) sensors are non-contact measurement devices that use laser beams to accurately detect the spatial position of a target. In previous studies, LiDAR sensors have been used to study droplet movement in the wingtip vortex of spraying aircraft (Hoff et al., 1989) to assess the spray aerosols drifting above orange orchards with the influence of meteorology parameters and atmospheric stability (Miller et al., 2003). Gregorio et al. (2015) developed an *ad hoc* LiDAR system for the measurement of pesticide spray drift, this system evaluates the amount of spray drift through laser signal strength. With this system, the optional spray drift reduction of hollow-cone nozzles was assessed (Gregorio et al., 2019). Currently, various types of LiDAR sensors are used for spray drift measurement. Commercial LiDAR technology is mature and highly available, exhibiting significant potential for broad and long-term applications in drift detection. Most commercial LiDAR sensors obtain plenty of distance values by scanning point clouds to construct target contours, which provides the possibility for the detection of dispersed drift droplets in space. A commercial 2D LiDAR sensor has been used to estimate drift measurement in vineyard spraying, where the detection results were compared with passive collector experiments to demonstrate the potential of 2D LiDAR for drift measurement of air-assisted sprayer (Gil et al., 2013).

This study aims to explore the feasibility of using a commercial 3D LiDAR sensor to assess spray drift. Spray drift tests with different working parameters were conducted in a wind tunnel, and the relationship between spray drift measurements obtained with LiDAR and passive collectors was analyzed.

## Materials and methods

### 3D LiDAR sensor

**Figure 1:**
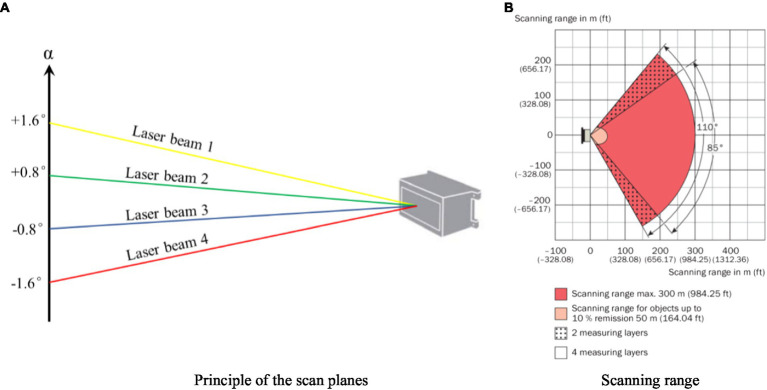
Scanning properties of the LiDAR sensor (Operation instructions of LD-MRS 3D LiDAR sensors, Sick AG, 2010). **(A)** Principle of the scan planes. **(B)** Scanning range.

The 3D LiDAR sensor used in this study was an outdoor four-layer scanner designed for harsh environments (model LD-MRS400001, Sick, Dusseldorf, Germany), with a long range of 300 m. The sensor adopted a four-line design to simultaneously emit four laser beams to form four stacked planes, with a scanning interval angle of 0.8° and a whole scanning angle of 3.2° (−1.6° to 1.6°) in the vertical direction (Figure 1A). In the horizontal direction, the sensor had a central scanning range of 85° for four scan planes, and the scanning range was extended between +35° and +50° or −50° and −60° to a total range of 110° (Figure 1B). The droplet detection was performed with laser beams emitted by the sensor in four stacked planes, where droplets impacted with the laser to form a drift cloud. Compared to single-wire LiDAR with one laser beam, this design ensures that more data signals are acquired in a scan procedure. The sensor had scanning frequencies of 12.5, 25, and 50 Hz. The available angular resolution was dependent on the scanning frequency, set to 0.125° or 0.25° under 12.5 Hz, 0.25° under 25 Hz, and 0.5° under 50 Hz. The sensor was connected to a computer *via* Ethernet or the RS232 serial port for configuration and data transfer of measurements. The specifications of the sensor are listed in Table 1.

**Figure 2:**
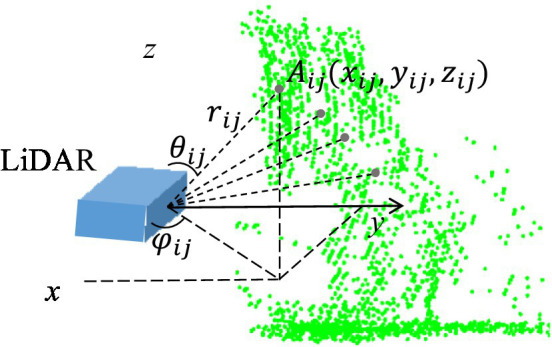
Schematic of the drift cloud scanned in the Cartesian coordinate system.

**Table 1:** 3D LiDAR sensor specifications.

Parameter	Technical indicators	Experiment settings
Wavelength (nm)	905	—
Laser class	1 (IEC 60825–1:2014)	—
Horizontal aperture angle (°)	110 (−60 ~ 50)	—
Vertical aperture angle (°)	3.2	—
Working range (m)	300	—
Scanning frequency (Hz)	12.5/25/50	25
Angular resolution (°)	0.125/0.25/0.5	0.25
Protection class	III	—
Enclosure rating	IP69K	—
Weight (kg)	1	—
Dimensions (mm)	94 × 165 × 88	—
Interface mode	RS-232/TCP/IP	—

The sensor had the multi-echo capability to gather and evaluate up to three echoes per transmitted laser pulse. As different objects form different echo voltages, the echo signals that may interfere with the reflected objects can be filtered by setting the threshold voltage. Therefore, the system was configured with a noise filtering function. The sensor also had high scanning sensitivity for objects with transparent properties, such as rain, fog, and glass, which ensured the feasibility of using the sensor to detect drift droplets.

### Data processing of drift points in space

SOPAS ET configuration software (V 02.18, Sick Sensor Intelligence) was used to manage the LiDAR sensor. Using this software, operators can configure and test measurement properties, analysis behavior, and output properties of the sensor as required. The sensor issued the original measured distance and angle information of drift droplets in reference to its coordinates. Initially, an angular coordinate system was constructed that contained each droplet spot scanned. Assuming that point A is a droplet in space (the *j*_th_ droplet in *i*-plane), its polar coordinates ( $r_{ij},\varphi_{ij},\theta_{ij}$ ) are expressed as:


(1) $$\left\{\begin{array}{c} r_{ij}=\left(RangeValue_{i}\left(j\right)\;\cdot \;scaleFactor\right)/1000\;\\ \varphi_{ij}=\left(startAngle_{i}+j\;\cdot \;angularResolution\right)/10000\\ \theta_{ij}=90^{{}^{\circ}}-\alpha_{i} \end{array}\right.$$

Where $r_{ij}$ is the actual distance between droplet A and the sensor, m; $\varphi_{ij}$ is the horizontal angle of droplet A; $\theta_{ij}$ is the vertical angle of droplet A; $RangeValue_{i}\left(j\right)$ is the original data output by SOPAS ET software; *i* represents the scan plane number between 1 ~ 4; scaleFactor is the factor by which the following RangeValue *s* can be brought to mm scale; $startAngle_{i}$ is the initial scan angle of the *i*-plane; angularResolution is the angular resolution in the horizontal direction; and $\alpha_{i}$ is the angle between the four planes in the vertical direction, with values of −1.6°, −0.8°, 0.8°, and 1.6°.

The scanned droplet point, in Cartesian coordinates, was reconstructed with MATLAB (R2018a, MathWorks Inc., Massachusetts). As shown in Figure 2, the coordinates of the droplet point A $\left(x_{ij},\;\;y_{ij},\;\;z_{ij}\right)$ are given by:


(2) $$\left\{\begin{array}{c} x_{ij}=r_{ij}\;\cdot sin\theta_{ij}\;\cdot \;\cos\varphi_{ij}\;\\ y_{ij}=r_{ij}\;\cdot \;\sin\theta_{ij}\;\cdot \sin\varphi_{ij}\;\\ z_{ij}=r_{ij}\cdot \cos\theta_{ij}\; \end{array}\right.$$

### Spray drift testing in a wind tunnel

**Figure 3:**
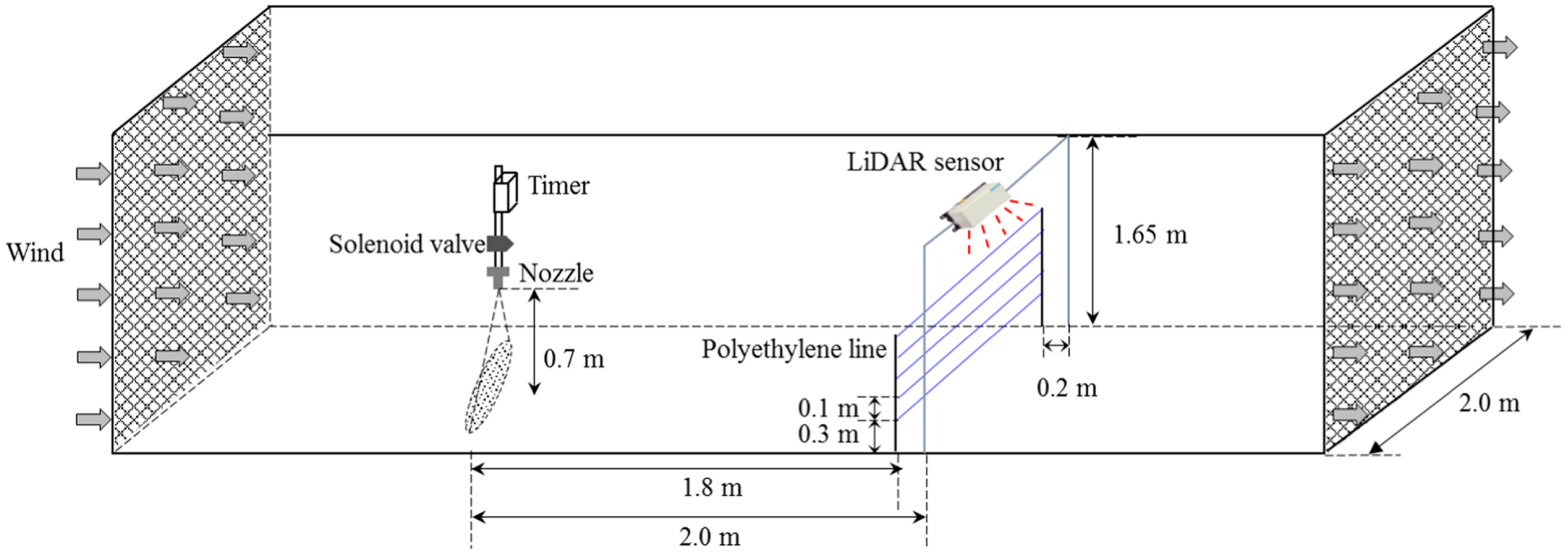
Construction of a test platform for measuring droplet drift in the wind tunnel.

Spray drift tests were conducted at the IEA-II wind tunnel at the National Experiment Station for Precision Agriculture, Beijing, China. A diagram of the wind tunnel is presented in Figure 3. This wind tunnel has been used in previous studies, such as Zheng et al. (2017); Zhang et al. (2019), and Tang et al. (2020). The wind tunnel consisted of an open-ended design, with a working section of 6.0 m length, 2.0 m width, and 2.0 m height. The wind tunnel applied an axial flow fan as the power source. Under the combined action of the rectifier and rectifying device, a uniform and stable wind field was generated. The adjustable range of the wind speed in the working section was 0.5 to 7 m/s; the turbulence was less than 0.3%, and the wind uniformity was less than 0.5%. The wind tunnel specifications fulfilled the requirements of the ISO 22856:2008 standard (ISO, 2008).

In this study, drift tests were conducted in strict accordance with ISO 22856:2008. A single and static nozzle was used, with the spray orientated at a right angle to the wind direction (Figure 3). The nozzle was fixed at a height of 0.7 m from the bottom of the wind tunnel. The selected test nozzles were a standard flat-fan spray nozzle ST11002, an air-inclusion spray nozzle IDK12002, and a hollow-cone nozzle TR8002. The spray patterns of the nozzles used have representative characteristics and are widely used (Nuyttens et al., 2007a; Peter et al., 2008; Torrent et al., 2019). A mixture of a water-soluble tracer and yellow tartrazine, with a concentration of 8 g/l, was used as the spray solution. To precisely control the spraying time, a timer was equipped upstream of the nozzle. When the spray time reached the preset value, the timer automatically switched the power off, and the spray system stopped. In this study, the spraying time was set to 20 s. The spray pressure was set to 0.2, 0.3, and 0.4 MPa, and the wind speed was set to 1 to 3 m/s with an interval of 0.5 m/s. During all tests, the temperature of the wind tunnel was 25°C, and the relative humidity was 36%.

Spray drift measurements were performed with both the LiDAR sensor and the passive collectors, following ISO 22866. Before the wind tunnel tests, the flow rates of the three nozzles used were measured by the weighing method. Furthermore, the droplet spectra were tested with a laser particle analyzer (HELOS-VARIO, Sympatec GmbH, Germany). In this study, *D*_*v*10_, *Dv*_50_, and *Dv*_90_ were measured, and relative span factor (RS), which represents a dimensionless indicator of the uniformity of the drop size distribution, was calculated according to equation (3); during the test, the nozzle was fixed at 0.5 m above the analyzer. The flow rates and droplet spectra under various working conditions are shown in Table 2. According to the experimental setup, a total of 60 spray drift tests were conducted.


(3) $$RS=\frac{D_{v90}-D_{v10}}{D_{v50}}$$

**Table 2:** Flow rate and droplet spectra of nozzles.

Nozzle model	Nozzle type	Pressure/MPa	Flow rate/L·min^−1^	*D*_*v*10_/μm	*D*_*v*50_/μm	*D*_*v*90_/μm	*RS*
ST11002	Flat-fan	0.2	0.65	71.84	159.74	279.15	1.298
		0.3	0.80	54.57	134.36	231.66	1.318
		0.4	0.92	51.05	124.97	206.76	1.246
IDK12002	Air-inclusion	0.2	0.65	142.49	327.19	594.33	1.381
		0.3	0.80	126.87	287.84	560.16	1.505
		0.4	0.92	109.04	251.20	514.90	1.616
TR8002	Hollow cone	0.2	0.65	64.70	140.88	232.12	1.188
		0.3	0.80	55.47	127.19	209.03	1.207
		0.4	0.92	47.35	115.39	196.56	1.293

Where, *RS* is the relative span factor; *D*_*v*10_, *D*_*v*50_, and *D*_*v*90_ are the maximum droplet diameter below which 10%, 50%, and 90% of the volume of the sample exists, respectively, *μ*m.

#### Sampling process using passive collectors

As shown in Figure 3, a vertical stainless-steel bracket was placed at a horizontal distance of 1.8 m from the nozzle in the downwind direction in the wind tunnel. Five polyethylene lines with a diameter of 2.0 mm were fixed horizontally across the bracket from 0.3 to 0.7 m, at 0.1 m intervals, to sample airborne drift droplets. The minimum height of 0.3 m was fixed to eliminate the impact of droplets bouncing and ground pollution on the test results. When the spraying finished, the polyethylene lines were collected into separate Ziploc bags, and the samples were stored in a dark box. After all the tests had finished, the polyethylene lines were brought to the laboratory for quantitative analysis. Five milliliters of deionized water was added to each Ziploc bag, and it was shaken sufficiently to fully elute and dissolve the tracer on the line surface. The absorbance of the eluate was measured using a visible light spectrophotometer (752 N INESA, Shanghai, China), and the amount of tracer droplets on the passive collector surface was calculated according to:


(4) $$\;\beta_{dep}=\frac{\left(Abs_{samp}-Abs_{blk}\right)\times V_{dil}\times 10^{3}}{Abs_{spray}}$$

Where, $\beta_{\mathrm{dep}}$ is the drift deposition volume on the passive collector surface in *μ*L; *Abs*_samp_ is the spectrophotometer absorbance value of the sample; *Abs*_blk_ is the absorbance reading of the blanks; *V*_dil_ is the volume of dilution liquid used to solute the tracer from the passive collector in mL; and *Abs*_spray_ is the spectrophotometer absorbance value of the spray mixture.

#### Spray drift measure algorithm using 3D LiDAR

The LiDAR sensor was fixed on the side of the wind tunnel closest to the vertical bracket. To ensure that the laser beam emitted by the sensor covered the polyethylene lines in the vertical array, the sensor was fixed 1.65 m above the wind tunnel floor, and the laser emitting surface faced downward. To prevent the passive collectors from blocking the laser beams, and considering the scanning planes of the sensor, the horizontal distance between the sensor and the vertical plane of the polyethylene line was set to 0.2 m. In the test, the scanning frequency and angular resolution of the sensor were set to 25 Hz and 0.25°, respectively. To gather more drift droplet points, four layers were used for the evaluation. The sensor was turned on before spraying, and the scanning measurements were initiated with the SOPAS ET software. The scanning time for each test was 1 min. The original data were then exported to the computer, and the drift droplet point was calculated according to equations (1) and (2).

To compare the measurements performed with the LiDAR sensor and the results obtained from the passive collectors, the number of drift points in five height intervals of 0.25–0.35, 0.35–0.45, 0.45–0.55, 0.55–0.65, and 0.65–0.75 m was calculated, corresponding to polyethylene lines at heights of 0.3, 0.4, 0.5, 0.6, and 0.7 m. Assuming the scanning point $A_{ij}(x_{ij},y_{ij},z_{ij}$ )satisfies equation (5), the cumulative number of drift points in the corresponding height interval should increase by increments of one.


(5) $$\left\{\begin{array}{c} x_{\min}\leq x_{ij}\leq x_{\max}\\ y_{0}+\left(k-1\right)\;\cdot \;\Delta d\leq y_{ij}<y_{0}+\left(k\right)\cdot \;\Delta d\\ z_{ij} \end{array}\right.$$

Where, $x_{\min}$ and $x_{\max}$ are the minimum and maximum values of the x-axis of the effective scanning area at $x_{\min}$ = − 1.0 m and $x_{\max}$ =1.0 m, respectively; $y_{0}\;$ is the minimum height of the effective scanning area, at $y_{0}$ =0.25 m; Δd is the height interval between adjacent lines, at Δd =0.1 m; and k is a constant, at *k* = 1, 2, 3, 4, and 5. The droplet points obtained by scanning in the z-axis direction are all valid; therefore, the *Z_ij_* is unlimited.

## Results

### Distribution of drift cloud and drift deposition in a vertical profile

**Figure 4:**
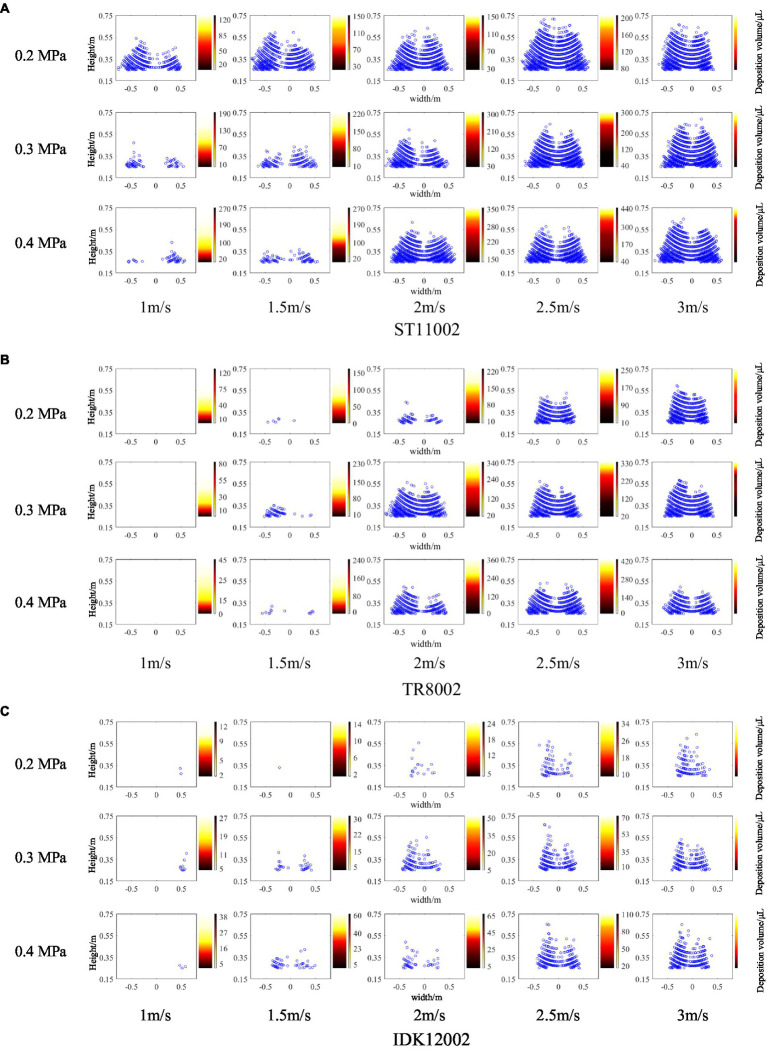
Drift points scanned by the LiDAR sensor (left of each panel) and drift deposition captured by passive collectors (right of each panel) for the three nozzles. In the strip plot for each combination, darker colors represent greater drift deposition. **(A)** ST11002. **(B)** TR8002. **(C)** IDK12002.

The number of droplet points at different height intervals scanned with the LiDAR sensor was counted, and the amount of tracer droplets deposited on the passive collectors was measured by a spectrophotometer. Figure 4 presents an overview comparison of the drift distributions obtained by the two methods. For each nozzle, a total of 15 panels were obtained under different working conditions, the left of each panel shows the drift points scanned by the LiDAR sensor, and the colored strip plot on the right side of the panel shows the deposition volume in vertical profile. The drift point cloud captured by the LiDAR sensor presents a triangular contour, where the distribution of droplets in the lower section is large and dense. As the height increases, the number of drift points tends to decrease, which is consistent with the results obtained from the passive collectors (from bottom to top, the color of the strip plot gradually fades). For the three nozzles used, the highest number of drift points was produced by the nozzle ST11002, followed by TR8002. The IDK120-02 nozzle had the least drift points, scanned under the same pressure and wind speed as the two other nozzles. The main reason for this finding is that large droplets formed in the air, limiting spray drift (Nuyttens et al., 2009; Vashahi et al., 2018). Under constant pressure, as the wind speed increases, the drifting droplets tend to be denser.

**Figure 5:**
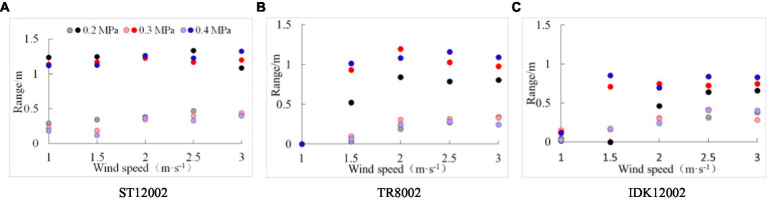
Width and height range of drift point distributions scanned by the LiDAR sensor for the three nozzles. The circles filled with solid color represent the width range in the horizontal direction and the circles filled with dotted point represent the height range in the vertical direction. **(A)** ST12002. **(B)** TR8002. **(C)** IDK12002.

The conventional sampling method is limited by the number and arrangement of samples, making it difficult to obtain the complete spatial distribution of drift droplets. In this section, the height and width ranges of the drift cloud under various operating parameters were calculated based on droplet point coordinates. As shown in Figure 5, the width range of nozzle ST11002 is higher than 1.0 m for all test conditions, which is significantly higher than that of nozzles TR8002 and IDK12002. Despite the spray angle of nozzle IDK12002 being 120°, which is higher than the other two nozzles as it is, affected by the larger droplets produced (Table 2), the width range is smaller than that of nozzles ST11002 and TR8002. In general, for the vertical direction, as the wind speed increases, the height range also increases, and there is little difference between the nozzles.

**Figure 6:**
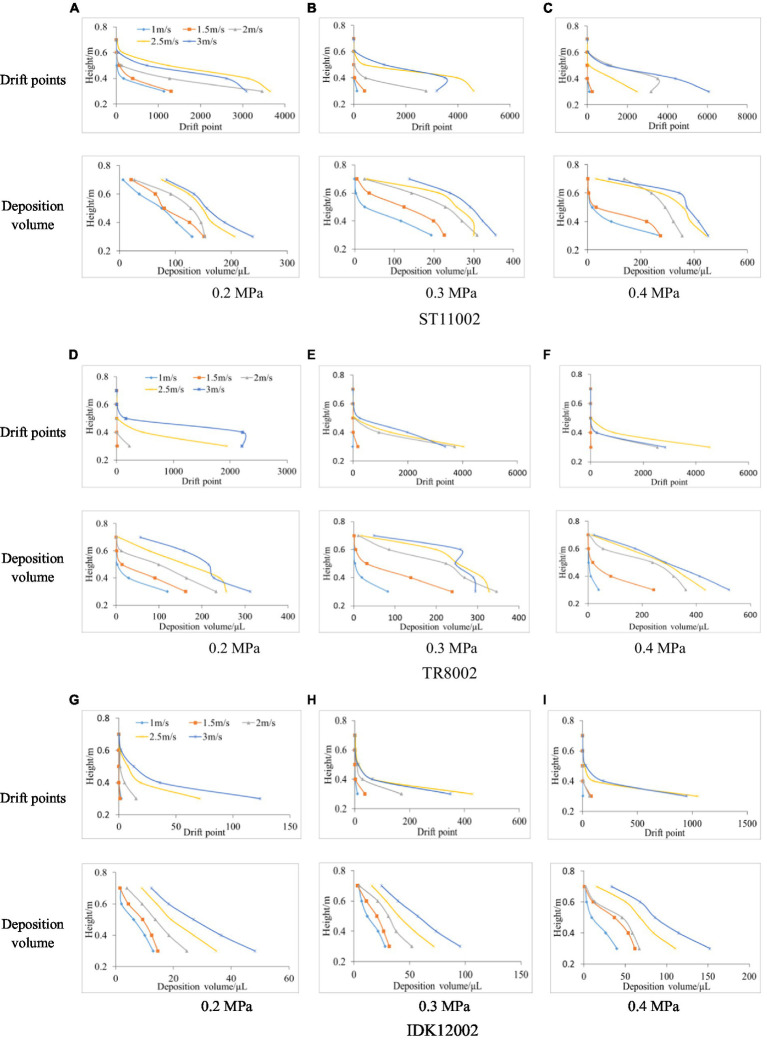
Spray drift obtained with passive collectors and LiDAR sensors at various heights for the three nozzles. **(A)** 0.2 MPa. **(B)** 0.3 MPa. **(C)** 0.4 MPa. **(D)** 0.2 MPa. **(E)** 0.3 MPa. **(F)** 0.4 MPa. **(G)** 0.2 MPa. **(H)** 0.3 MPa. **(I)** 0.4 MPa.

Figure 6 presents the drift deposition volume and the corresponding scanning points for the vertical profile. In general, the spray drift obtained by the two methods decreases gradually as the height increases. Compared with the passive collector sampling method, the LiDAR technique does not exhibit high capture sensitivity, especially at greater heights. For example, at a pressure of 0.2 MPa, the drift deposition volume of nozzle ST11002 was 6.255, 20.943, and 26.405 μl for wind speeds of 1.0, 1.5, and 2.0 m/s, respectively, while the LiDAR failed to scan any droplet in the height range of 0.65 to 0.75 m. The differences may be a result of the difficulties in the laser beam impacting the low-density point cloud due to a reduced number of drift droplets.

Laser beams emitted by LiDAR sensors are in a divergent radiation mode, implying that the scanning results are affected by the frequency and angular resolution, which makes it difficult to make the actual number of droplets in the space completely consistent with the returned effective laser signal. In this study, through the comparative analysis of the drift deposition volume and droplet points of the 60 tests, it was found that when the deposition volume was less than 50 μl, LiDAR is prone to invalid scanning, that is, it is difficult to get more feedback laser signal.

### Correlation analysis between LiDAR and indirect methods

**Figure 7:**
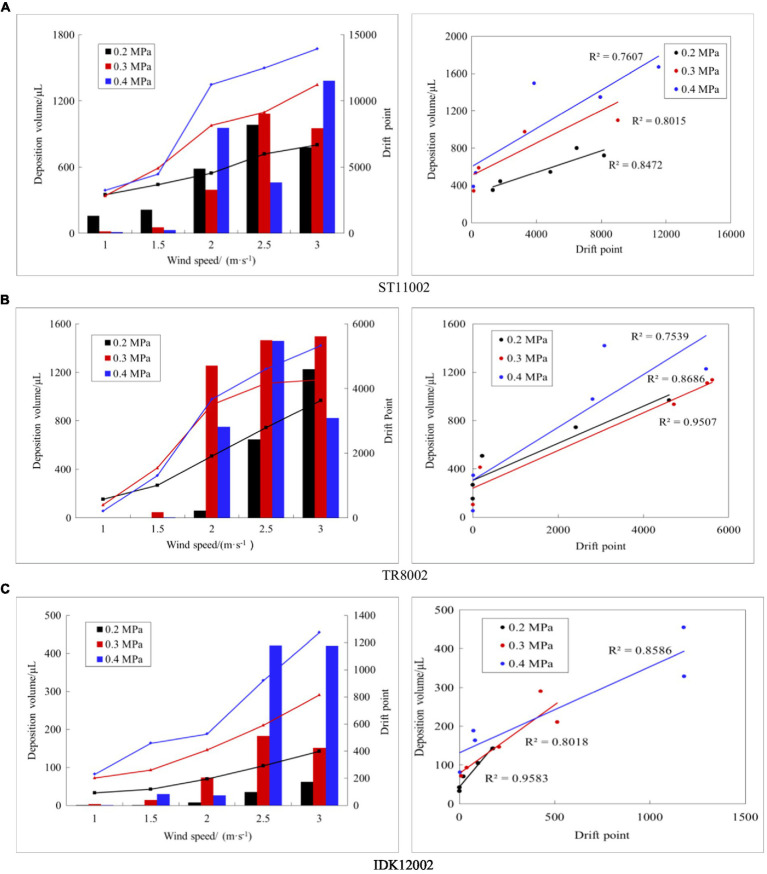
Correlation analysis of drift points and deposition volume for the three nozzles. The left panel shows the drift points and deposition under various working conditions (line represents deposition volume, column represents drift points), and the right panel shows the correlation between the two methods. **(A)** ST11002. **(B)** TR8002. **(C)** IDK12002.

The drift deposition and the droplet points through the vertical profile were processed further, and a correlation analysis was performed (Figure 7). For the three types of nozzles used, the drift deposition volume for passive collectors gradually increased with increasing wind speed. When the wind speed exceeded 1.5 m/s, the deposition volume increased gradually with an increase in spray pressure (0.4 > 0.3 > 0.2 MPa). The drift points captured by the LiDAR sensor did not show a same regularity as the deposition volume. At 1–2 m/s, the number of drift points gradually increased with an increase in wind speed, while the point number at 2.5 m/s may be less than 3 m/s. For example, for nozzle ST12002 at a spray pressure of 0.4 MPa, the number of drift points at a wind speed of 2.5 m/s was 9,024, which is higher than 7,925 drift points at a wind speed of 3 m/s. The possible reason is that the higher movement speed of droplets affects the capture ability. The IDK12002 nozzle has significantly lower deposition and drift points than the ST11002 and TR8002 nozzles. In this case, 3D LiDAR measurement can classify the drift performance of the conventional nozzle and the anti-drift nozzle.

Through the correlation analysis of 3D LiDAR and the indirect method, it was found that the drift point number captured by 3D LiDAR generally has a good correlation with the deposition volume from passive collectors, with the coefficients of determination (*R*^2^) of the three nozzles being greater than 0.75. Among the three nozzles, IDK122002 with less spray drift and larger droplet size has the best correlation, and the minimum *R*^2^ is 0.80 under the three spray pressure settings. In terms of spray pressure, the *R*^2^ of nozzles ST11002 and IDK12002 showed a decreasing trend with an increase in the spray pressure. When the spray pressure increased, the droplet size decreased (Table 2), and the amount of drifting droplets increased. The laser beam impacted the droplets directly in front of LiDAR, but a few laser beams failed to capture droplets further away from the LiDAR sensor because of the blocking effect of the droplets ahead.

### *Wt* analysis of the influence of spray parameters on 3D LiDAR drift assessment

Changes in spray parameters can affect the drift deposition volume and drift points captured by LiDAR sensors. Through the previous analysis, it was found that the scanning accuracy of 3D LiDAR is different under different droplet size spectra, flow rates, and wind speed conditions. Understanding the influence of these factors can provide support for the rational use of 3D LiDAR to evaluate spray drift. In this study, SPSS software was used to analyze the linear relationship between drift points, deposition volume, and the coefficients of determination *R*^2^ value of the two methods with the flow rate, *D*_*v*50_, *RS,* and wind speed. The corresponding coefficients were calculated, as shown in Table 3. The larger the absolute value of the coefficient, the greater the influence of the parameter on the result.

**Table 3:** Coefficients of spray parameters, according to the linear analysis of drift points, deposition volume, and *R*^2^.

Spray parameter	Flow rate	*D*_*v*50_	*RS*	Wind speed
Drift deposition volume measured by passive collector	0.303	−0.327	−0.312	0.571
Drift points scanned by LiDAR	−0.062	−0.497	0.030	0.580
*R*^2^ of LiDAR and indirect method	−0.219	0.715	−0.519	—

**Figure 8:**
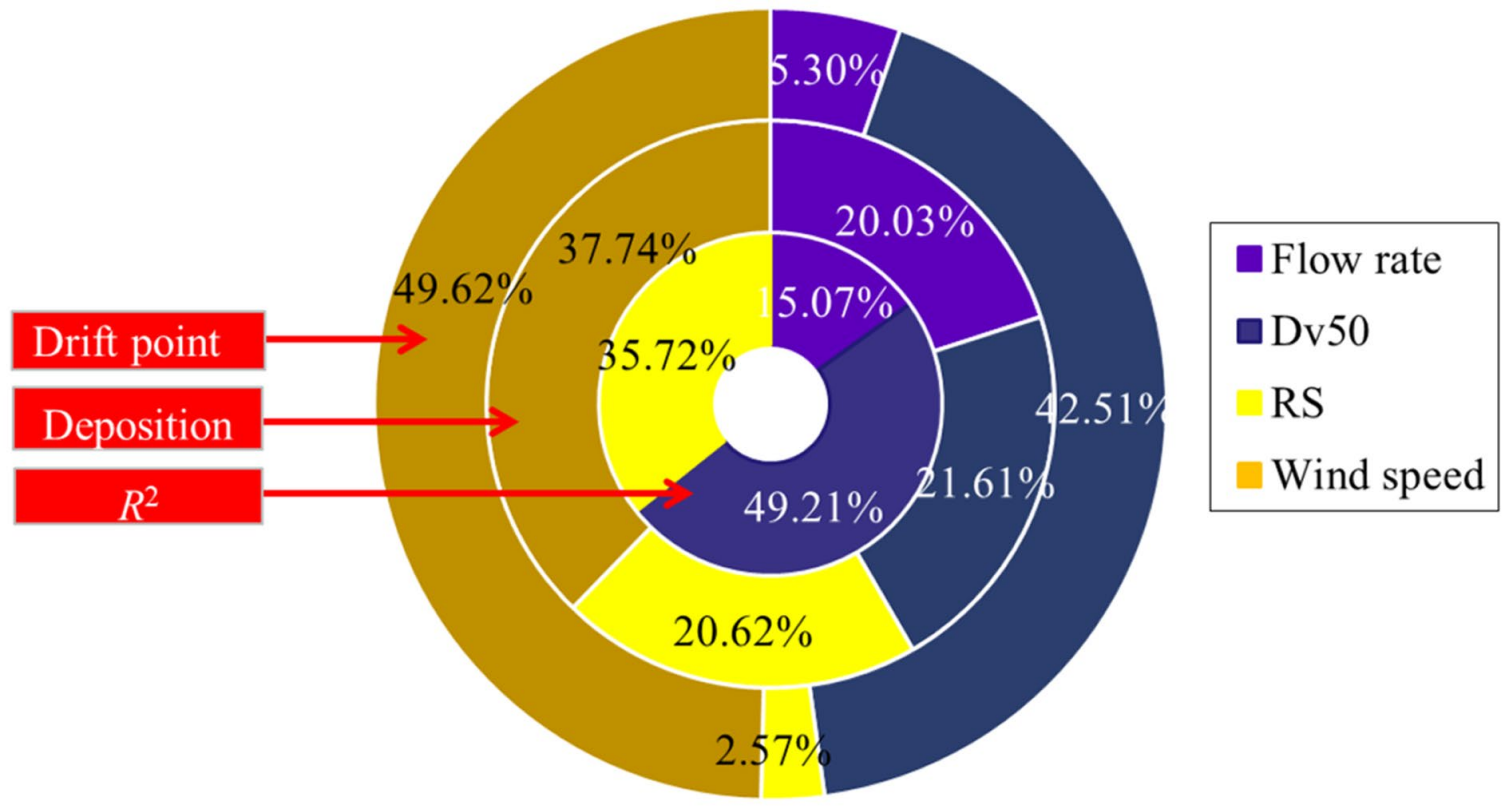
Influence weights of spray parameters on drift assessment.

The influence weights of each parameter were calculated based on the data in Table 3, as shown in Figure 8. The wind speed had the greatest influence on the sampling method by passive collectors with a ratio of 37.74%, with the flow rate, *D*_*v*50,_ and relative span factor (*RS*) being equally weighted. For the drift point scanned by LiDAR, the influence of wind speed and *D*_*v*50_ accounts for a great proportion, and their influence weights are 49.62% and 42.51%, respectively. For the *R*^2^ values of the two methods, the droplet spectrum had a greater influence, and the weight ratio of *D*_*v*50_ and *RS* was more than 80%.

## Discussion

In pesticide application process, fine droplets may drift to the non-target area and cause serious environmental and public health problems, including damage to the adjacent crops sensitive to chemical agents, river contamination, and risk to the health of humans and animals (Nuyttens et al., 2010). At present, spray drift is unavoidable. However, we can optimize the chemical application technology by means of drift evaluation, i.e., nozzle selection, operating parameters adjustment, and suitable working surroundings. Traditional spray drift experiments are complex, time-consuming, and labor-intensive. Therefore, there is a strong demand for an efficient and convenient alternative drift measurement method.

In this study, exploratory work was conducted to demonstrate the capacity of a commercial 3D LiDAR sensor to evaluate spray drift generated by different nozzle types, spray pressures, and wind speeds. LiDAR measurements were compared with those obtained with the indirect method using passive collectors. Firstly, the coordinates of the drift points scanned by the LiDAR sensor were converted to obtain the point clouds in the vertical profile, intuitively observing the drift droplet distribution (Figure 4). This is difficult to achieve with traditional indirect methods. The drift width and height ranges under various working conditions were calculated through point cloud coordinates (Figure 5). The results can provide a point of reference for setting the size of anti-drift obstacles (De Schampheleire et al., 2009).

The drift points in various height intervals were counted. It was assumed that each height interval was 0.1 m (with the polyethylene line as the center, the upper and lower heights were both 0.05 m). Accordingly, the drift points and deposition volume for passive collectors were compared (Figure 6). Although the LiDAR sensor used a higher scanning frequency of 25 Hz, few droplets impacted the laser beam owing to the lower droplet density at a higher height interval (0.7 m). LiDAR sensor determines drift from the reflected signal of a laser beam impacting a droplet, while it is difficult to equate a laser feedback signal with a droplet, and laser beam impacts are sensitive to droplet density or drift mass. In addition, by correlating the drift points with the deposition volume obtained by the indirect method (Figure 7), it is observed that the nozzle IDK12002 has a better correlation between 3D LiDAR measurements and the indirect method, and the lower spray pressure with less drift appears to be more conducive to drift evaluation with 3D LiDAR. Conversely, Gil et al. (2013) conducted a study using a commercial 2D LiDAR sensor to evaluate the spray drift of orchard sprayers, the results indicate a bad ability of the 2D LiDAR sensor to evaluate spray drift in case of sparse drift cloud with air-inclusion nozzles. The droplet density or drift mass suitable for LiDAR measurement is likely to have a threshold range, beyond which the detection accuracy will be reduced. By comparing all the test data in this study, we found that when the deposition volume was less than 50 μl, 3D LiDAR is prone to invalid scanning.

The drift deposition volume from passive collectors gradually increased with increasing wind speed, while the number of drift points measured by LiDAR does not follow the same law. For example, for nozzle ST12002 at a spray pressure of 0.4 MPa, the number of drift points at a wind speed of 2.5 m/s was 9,024, which is higher than 7,925 drift points at 3 m/s (Figure 7). This phenomenon may be caused by excessive droplet density or by changes in wind speed. When the wind speed is higher, the fine droplets pass through the vertical profile at a higher speed, and either the emitted laser beam fails to perfectly impact the droplets or the high-speed droplets cause part of the energy loss, implying that the reflected signal strength cannot reach the LiDAR system identification threshold.

Through the *wt* analysis, it was found that the *D*_*v*50_ and *RS* have a great influence on R^2^ of LiDAR and indirect method (Figure 8). The droplet spectrum also indirectly affects the droplet density in the detection area. In order to reduce the detection distortion caused by laser beam occlusion and laser beam emission angle resolution, an appropriate detection area needs to be identified in advance. Although the current LiDAR sensor has a maximum detection distance of 300 meters or more, in actual spray drift evaluation, only a small plane (e.g., 1 × 1 m) close to the LiDAR may be selected as the sampling zone. This selected plane needs to be determined by experiments so that LiDAR can restore the spatial distribution of droplets most realistically.

In addition to the factors of the spray drift flux mode, the spray drift measurement with LiDAR in field maybe faces the challenges such as the impact of higher-intensity sunlight, dust suspended in the air, and ambient temperature on the performance of LiDAR. Gregorio et al. (2019) confirmed that spray drift measurement distortion maybe occured because of the presence of air-suspended dust based on the LiDAR system developed. Nowdays, the research on LiDAR detection performance in agriculture mainly focuses on sensing geometric characterization of canopy and obstacle in agricultural activities (Lee and Ehsani, 2008; Rosell and Sanz, 2021). Commercially available LiDAR sensors are expected to be a practical tool for drift assessment. However, the current research depth and breadth are not enough. It is essential to carry out subsequent research combined droplet characteristics, drift point cloud spatial distribution, application scenarios, and environmental conditions, to determine the optimal conditions for LiDAR measurements such as droplet density ranges, LiDAR Settings, and environmental conditions.

## Conclusion

3D LiDAR sensors provide a fast and efficient detection method for evaluating the drift performance of different types of nozzles and spraying techniques. Through non-contact scanning, the spatiotemporal distribution plots of drifting droplets can be provided, and the influence of environmental characteristics on the spatial transport of drifting droplets can be evaluated. Compared with the traditional method of using passive collectors, LiDAR technology significantly reduces time and labor cost, as well as the operator’s exposure to chemical pesticides.

In general, a good correlation was observed between the drift deposition with passive collectors and the drift points scanned by 3D LiDAR. This non-contact sensing method has shown potential for evaluating spray drift characteristics of nozzles under different working conditions. However, it is difficult to equate a laser feedback signal with a droplet, the droplet detection performance of commercially available 3D LiDAR sensors is limited by sensitivity to droplet density. It can be inferred that the effectiveness of LiDAR on droplet detection has certain threshold requirements for droplet density, knowing the optimal droplet density range can greatly improve the detection accuracy of LiDAR. Also, the droplet spectrum and movement speed may be other important factors, which affect the strength and quantity of the reflected signal of a laser beam impacting droplet. In this study, IDK12002 shows the best correlation between 3D LiDAR measurements and the indirect method, and the lower spray pressure with less drift and larger droplet size appears to be more conducive to drift evaluation with 3D LiDAR. Further research would be arranged to investigate the influence of droplet size and movement speed on detection results, and clarify the maximum and droplet density threshold range allowed by 3D LiDAR detection.

## Data availability statement

The raw data supporting the conclusions of this article will be made available by the authors, without undue reservation.

## Author contributions

LL: conceptualization, methodology, writing—original draft, and funding acquisition. RZ: methodology, supervision, writing—review and editing, and funding acquisition. LC: software, formal analysis, and writing—review and editing. BL: resources, software, data curation, and investigation. LZ: software and data curation. QT, CD, and ZZ: resources, validation, and investigation. AH: methodology. All authors contributed to the article and approved the submitted version.

## Funding

This work was supported by National Key R&D Program of China (2019YFD1101102-3), the National Natural Science Foundation of China (32071907), and Youth Research Fund of BAAFS (QNJJ202009).

## Conflict of interest

The authors declare that the research was conducted in the absence of any commercial or financial relationships that could be construed as a potential conflict of interest.

## Publisher’s note

All claims expressed in this article are solely those of the authors and do not necessarily represent those of their affiliated organizations, or those of the publisher, the editors and the reviewers. Any product that may be evaluated in this article, or claim that may be made by its manufacturer, is not guaranteed or endorsed by the publisher.
